# Changes in the Proteasome Pool during Malignant Transformation of Mouse Liver Cells

**Published:** 2010-04

**Authors:** T.M. Astakhova, G.V. Delone, Yu.V. Lyupina, E.B. Abramova, I.V. Uryvaeva, N.P. Sharova

**Affiliations:** Koltsov Institute for Developmental Biology, Russian Academy of Sciences

**Keywords:** immunoproteasomes, 19S proteasome activator, chymotrypsin–like activity of proteasomes, Western blot analysis, nodular regenerative hyperplasia of the liver, adenoma, hepatocellular carcinoma, mouse liver

## Abstract

Multiple forms of proteasomes regulate cellular processes by destroying proteins or forming the peptides involved in those processes. Various pathologies, including carcinogenesis, are related to changes in the functioning of the proteasome forms. In this study, we looked at the changes in the pool of liver proteasomes during nodular regenerative hyperplasia and formation of adenoma and hepatocellular carcinoma in mice treated with Dipin, followed by partial liver resection. The relative content of various proteasome forms was determined using Western blot analysis. The chymotrypsin–like activity of proteasomes was assessed from the hydrolysis of the commercial Suc–LLVY–AMC substrate. It was found that changes in the proteasome pool appeared already during the formation of diffuse nodules, the changes being the increased expression of the X(β5) constitutive subunit and the LMP7(β5i) and LMP2(β1i) immune subunits, accompanied by the increase of the total proteasome pool and the decrease in the chymotrypsin–like activity. These changes were more pronounced in hepatocellular carcinoma. The content of the total proteasome pool and the LMP2(β1i) immune subunit and the chymotrypsin–like activity in adenoma were intermediate compared to those in the samples of liver with diffuse nodules and carcinoma. In addition, the level of the Rpt6 subunit present in the 19S proteasome activator was increased in carcinoma. Our results indicate that nodular regenerative hyperplasia and adenomatosis may be stages preceding carcinogenesis. We also conclude that there is a need to find signalling pathways that change the expression of various proteasome subunits during carcinogenesis. The 19S proteasome activator, which is overexpressed in malignant tumours, can be a promising target for the development of new anticancer drugs.

## INTRODUCTION

Understanding the molecular mechanisms underlying the malignant transformation of cells is of ever vital importance. The new protein hydrolysis system discovered in the 1980s involving proteasomes and affecting all cellular processes provided a new impulse to the studies of the mechanisms of mammalian cell malignant transformation. Proteasomes, multisubunit multiproteinase protein complexes, are present in mammalian organs and tissues in a multitude of forms of different structures and physiological functions [1–4]. Proteasomes can be divided into two groups—constitutive proteasomes and immunoproteasomes—depending on the nature of their active protease subunits. The constitutive proteasomes contain two of each of the X(β5), Y(β1) and Z(β2) subunits, possessing chymotrypsin–like, caspase–like, and trypsin–like activity, respectively. The immunoproteasomes contain the LMP7(β5i), LMP2(β1i), and LMP10(β2i) immune subunits instead of the above–mentioned protease active subunits of the constitutive proteasomes. When foreign proteins are hydrolysed by immunoproteasomes, the amount of antigen epitopes formed is several times higher. The antigen epitopes are capable of incorporating into the Bjorkman gap of the major histocompatibility complex class I molecules for further presentation to T lymphocytes. In addition, immunoproteasomes participate in the regulation of the differentiation and proliferation of some cell populations, perhaps, by producing biologically active peptides [5, 6]. They are also an essential part of the signalling pathway responsible for the quenching of oxidative stress [7].

Both constitutive proteasomes and immunoproteasomes form 26S and 20S proteasome pools [3]. The 26S proteasomes consist of the 20S proteolytic core particle and one or two of the 19S regulatory particles responsible for binding to ubiquitinated target proteins, the unfolding of those proteins, and directing them into the proteolytic chamber. Thus, the 26S proteasomes regulate cellular processes by degrading proteins or forming the peptides involved in those processes.They also trigger the reactions associated with the T–cell immune response. The 26S proteasomes are usually dependent on ATP and ubiquitin. The 20S proteasomes, on the contrary, degrade proteins and short peptides independently of ATP and ubiquitin. The number of proteins identified as substrates of 20S proteasomes increases every year; these include, for example, proteins with a damaged tertiary structure [8] and some virus proteins [9, 10].

The functions of proteasomes are very diverse, and determining the changes occurring in the proteasome pool during malignant cell transformation is important for understanding the transformation mechanism, as well as for finding new targets for anticancer therapy among the multiple forms of proteasomes. The scarce published data on this matter concern the comparison of separate proteasome form contents in malignant and control cells [11–16]. Information on how the proteasome pool functions during the growth of nonmalignant and malignant tumours could shed light on some of the mechanisms of cell transformation into the malignant state. The aim of this study was to determine the changes in the proteasome pool during the growth of nonmalignant and malignant tumours using the same model animals. We used a previously developed model to induce malignant transformation of liver cells in mice CBA/Lac x BL/6 F1 by alkylating drug, Dipin, followed by partial liver resection [17, 18]. Dipin causes irreparable damage of the genetic material in hepatocytes leading, after mitoses stimulated by partial liver resection, to chromosome breakage and rearrangement. The cells damaged in such a way are not viable, and they eventually die. The parenchyma is regenerated by means of activation of stem cells and clonogenic growth of neoplastic hepatic nodules, which coalesce and displace the original degenerating hepatocytes and form new tissue. This nodular regenerative hyperplasia is diffuse in nature, but, eventually, the separate nodules can progress and give rise to large adenomas and hepatocarcinomes. In this paper, we report on the comparative study of the chymotrypsin–like activity and total proteasome pool content, as well as the 26S proteasome and immunoproteasome contents, in the intact liver and induced nonmalignant and malignant liver tumours.

## EXPERIMENTAL

**Reagents**. The following reagents were used: Suc–LLVY–AMC and MG132 (Sigma, USA), anti–β–actin mouse mAb (Santa Cruz, Germany), anti–Rpt6 and anti–α1,2,3,5,6,7 mouse mAb, anti–X(β5), anti–LMP7(β5i) and anti–LMP2(β1i) rabbit pAb (Biomol, UK), anti–nNOS rabbit pAb (Abcam, UK), and ECL kit, Hybond–ECL nitrocellulose membranes and peroxidase conjugated antibodies to mouse and rabbit IgG (Amersham Biosciences, UK).

**Animals**. Male mice CBA/Lac x BL/6 F1, three months old, 20–22 g weight, were used in the study. Dipin at 60 microgram per 1 g of weight was injected to a group of male mice. A standard partial liver resection operation (up to 70 %) was performed according to a previously developed procedure [17]. Mice with intact liver and mice subjected to partial liver resection were used as controls. After 12 months, the livers of the control and test animals were studied.

**Histological study of the liver**. The fragments of the liver and large tumour nodules were fixed in 10% formalin. The fixed material was processed following the standard procedure: 5–micron–thick sections were prepared after paraffin embedding. After removing the paraffin, the specimens were H&E stained, embedded in balsam, and analysed with Olympus AHBT3 optical microscope.

**Preparation of clarified homogenates of liver and tumour fragments**. All procedures were performed at 0–4°С. Liver and tumour fragments were washed with a standard sodium phosphate buffer, dried, weighed, and homogenised (glass–glass, homogeniser Braun Melsungen, Germany) in a buffer containing 50 mM Tris–HCl (pH 7.5), 100 mM NaCl, 1 mM EDTA, 1 mM dithiothreitol, 10% glycerine, 5 mM MgCl_2_, 1 mM ATP, 10 mM Na_2_S_2_O_5_, leupeptin (0.5 µg/ml), pepstatin (1 µg/ml) and aprotinin (1 µg/ml) at 1 : 3 ratio. The homogenates were centrifuged at 10,000 g for 30 min. The supernatants (clarified homogenates) were used in the studies. The protein concentration in the clarified homogenates was determined by the Lowry method [19].

**Determination of proteasome activity**. The proteasome activity was determined by measuring the hydrolysis of the Suc–LLVY–AMC fluorogenic oligopeptide, which is a substrate for proteasome chymotrypsin–like sites [20]. The reaction mixture contained 20 mM Tris–HCl (pH 7.5), 1 mM dithiothreitol, 30 µM Suc–LLVY–AMC, 5 mM MgCl_2_, and 1 mM ATP. In order to eliminate the contribution of the proteolytic activity of impurities, 10 µM MG132 (inhibitor of the proteasome chymotrypsin–like sites) was added to some samples. The reaction was carried out at 37 °С for 20 min after adding 0.5–2 µl of clarified homogenate (to a total volume of 100 µl), and the reaction was stopped with 1% SDS. The product mixture was measured in a fluorimeter with excitation and emission at 380 and 440 nm, respectively. The difference between the total and residual activity in the presence of MG132 was calculated. The activity was expressed as nanomol of Suc–LLVY–AMC hydrolysed in 20 min by proteasomes contained in 100 µl of clarified homogenates.

**Western blot analysis**. Western blot analysis was used to determine the relative content of proteasome subunits, nNOS, and β–actin in the clarified homogenates. Gel electrophoresis of the proteins from clarified homogenates was performed in 10–13% PAA gel in the presence of SDS (5 µl per lane, 120–148 µg of protein). The polypeptides were transferred from the gel to a nitrocellulose membrane using the semi–dry method. The membrane was incubated for 2 hours at 20 °С in TNT buffer (10 mM Tris–HCl (pH 7.5), 150 mM NaCl, 0.1% Tween–20), and then for 1 hour in TNT buffer containing 2–5% of non–fat milk and mouse mAb to β–actin (1 : 200) or to Rpt6 (1 : 2500), or to α1,2,3,5,6,7 (1 : 2500) (or rabbit pAb to nNOS (1 : 500) or to X(β5), or to LMP7(β5i), or to LMP2(β1i) (1 : 1250)). The membrane was washed several times with TNT buffer and incubated for 1 hour in TNT buffer containing 2–5% of nonfat milk and peroxidase conjugated antibodies to mouse (or rabbit) IgG (1 : 2500). Then, the membrane was washed with TNT buffer and analysed using the ECL kit following the standard procedure.

ImageJ software was used for image processing. The relative content of proteins in the clarified homogenates was determined by measuring the density of corresponding bands on the X–ray film, using previously prepared calibration plots of density vs. analysed protein content. Further experiments were carried out within the range of protein concentrations for which the calibration plot showed linear behaviour.

**Statistical analysis**. The data are presented as average ± confidence interval (*δ*): *δ* = ± *tσ n*^–0.5^, where *t* is the Student’s criterion value at significance level *p* < 0.05, *σ* is the standard error, and *n* is the number of experiments.

## RESULTS AND DISCUSSION

**Fig. 1:**
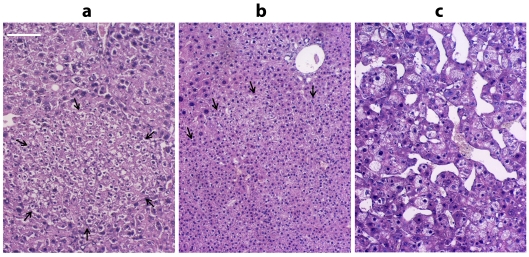
Hepatic tumors developed under chronic regenerative liver condition in the 12 months after treatment with Dipin followed by partial liver resection. a – microadenoma nodule consisting of small hepatocytes with diploid nuclei. b – large hepatocellular adenoma lacking typical liver lobule and vasculature structure. c – trabecular hepatocellular carcinoma with cytomegaly, anomalous trabecular and sinusoid structure. H&E staining. Arrows indicate tumour boundaries. Scale bar 100 microns

**Histological study of mouse liver**. The results of mouse liver histological study performed 12 months after Dipin injection and partial liver resection are presented in Fig. 1. Multiple nodules (benign tumours, microadenomas) were revealed in the liver tissue (Fig. 1 a); they formed during the diffuse nodular regenerative hyperplasia of hepatocytes. In addition, we detected large benign tumours, adenomas, (Fig. 1 b) and malignant tumours whose biological properties corresponded to hepatocellular carcinoma of the trabecular type (Fig. 1 c) [21]. We performed a comparative study of the chymotrypsin–like activity of the total proteasome pool and the content of various proteasome subunits in the tumour samples and liver fragments with diffuse nodular hyperplasia versus liver samples of the control animals.

**Technical notes on the study of changes in the proteasome pool during tumourogenesis**. The relative content of the total proteasome pool in the samples was studied by Western blot analysis using antibodies to α1,2,3,5,6,7 subunits present in all proteasome forms. In a similar way, we determined the relative content of the 26S proteasomes using antibodies to the Rpt6 subunit contained in the 19S particles of the 26S proteasomes, as well as the relative content of the X(β5), LMP7(β5i), and LMP2(β1i) proteolythic subunits, using corresponding antibodies.

**Fig. 2:**
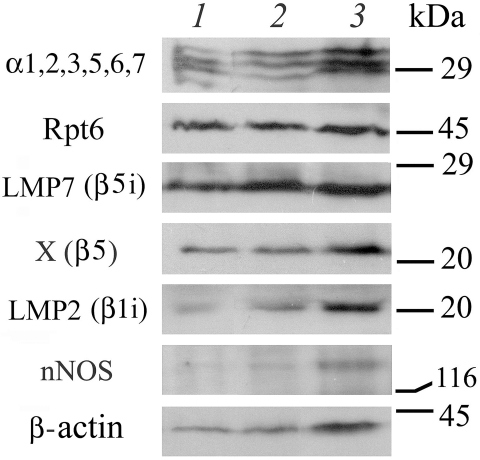
Western blot analysis of proteins in the clarified homogenates of the intact liver (1), liver with diffuse nodules (2), and hepatocellular carcinoma (3) using antibodies to proteasome subunits α1,2,3,5,6,7, Rpt6, LMP7(β5i), X(β5), and LMP2(β1i), nNOS and β-actin. Markers: carboanhydrase (29 kDa), ovalbumin (45 kDa), trypsin inhibitor (20 kDa), and β-galactosidase (116 kDa)

In addition to measuring the concentration of proteasome subunits in the clarified homogenates of liver and induced tumours, we studied the concentration of total proteins and β–actin, which are normally used for standardisation of the activity and concentration of proteins in tissues. The total protein content in the clarified homogenate of hepatocellular carcinoma was slightly—but reliably—lower than that in the clarified homogenate of the intact liver (Table 1). At the same time, the β–actin content was significantly higher in the hepatocellular carcinoma sample (Fig. 2, Table 1). This meant that in this study both these parameters could not be used as an internal reference for the standardisation of the protein properties. The more appropriate method would be to compare the activity and concentration of proteasome subunits in the control mice liver and hepatic tumours normalised to the raw tissue weight.

**Table 1:** Chymotrypsin–like activity of proteasomes and the content of the proteasome subunits, nNOS, β–actine, and total protein in the clarified homogenates of mouse liver and induced liver tumors.

Parameter		Value in the clarified homogenate of			
		intact liver	liver with hepatic nodular hyperplasia	adenoma	hepatic carcinoma
Chymotrypsin–like activity of proteasomes in a 100 β–l sample (nanomol Suc–LLVY–AMC)		18.6 ± 1.3	13.7 ± 0.5	11.2 ± 0.5	5.3 ± 0.3
Content of proteasome subunits (%)
	α1,2,3,5,6,7	100 ± 4	135 ± 3	147 ± 2	220 ± 6
	Rpt6	100 ± 3	98 ± 3	101 ± 5	150 ± 6
	X(β5)	100 ± 3	125 ± 5	–	210 ± 8
	LMP7(β5i)	100 ± 5	150 ± 4	170 ± 5	169 ± 7
LMP2(β1i)	100 ± 3	200 ± 7	290 ± 17	400 ± 15
nNOS content (%)		low	100 ± 3	–	450 ± 13
β-actin content (%)		100 ± 2	102 ± 3	97 ± 8	270 ± 7
Protein concentration (mg/ml)		29.5 ± 0.9	26.5 ± 1.1	26.0 ± 0.7	24.0 ± 0.8

A 100% level of proteasome subunits and β-actin correspond to their content (optical density of bands) in the clarified homogenate of the intact liver; A 100% level of nNOS correspond to its content in the clarified homogenate of the liver with hepatic nodular hyperplasia. The data are represented as the average value ± δ. For each data point, p < 0.05, n ≥ 5.

As an additional reference, we used liver regenerated after the partial resection, which was not treated with Dipin. For the clarified homogenates of that liver and the intact liver, no difference was found in the proteasome chymotrypsin–like activity, nor in the content of all studied proteasome subunits, β–actin, and total protein (data not shown).

**Differences and similarities in the change in the proteasome pool during benign and malignant tumour formation**. The changes in the proteasome pool appear as early as when diffuse nodules are being formed, manifested by the increase of the total proteasome pool and the expression of the X(β5) constitutive subunit and the LMP7(β5i) and LMP2(β1i) immune subunits (Fig. 2, Table 1). The extent to which the expression of these subunits is increased is demonstrated by the following pattern: LMP2(β1i) > LMP7(β5i) > X(β5). The increase in the LMP2(β1i) subunit content is equal to that of the third immune subunit LMP10(β2i), since they are always incorporated into proteasomes together, whereas the LMP7(β5i) immune subunit can be incorporated into proteasomes independently from the other two [22, 23]. The increase in the content of the subunits studied in the total proteasome pool was accompanied by a decrease in the total pool activity with respect to the Suc–LLVY–AMC oligopeptide hydrolysed by the chymotrypsin–like sites of the X(β5) constitutive subunit and the LMP7(β5i) immune subunit (Table 1).

The formation of a malignant tumour caused an even stronger decrease in the proteasome chymotrypsin–like activity, as well as a stronger increase in the total proteasome pool and the content of the immune subunits and the X(β5) constitutive subunit (Fig. 2, Table 1). The pattern of the increase of these subunits was different: LMP2(β1i) > X(β5) > LMP7(β5i). It should be noted that although the content of the LMP7(β5i) immune subunit in the hepatocellular carcinoma was higher than that in the control liver tissue and the liver fragments with diffuse nodules, it was the same as that in the adenoma. The content of the total proteasome pool and the LMP2(β1i) immune subunit, and the chymotrypsin–like activity in the adenoma were at an intermediate level compared to those in the samples of liver with diffuse nodules and hepatocellular carcinoma (Fig. 2, Table 1). These results indicate that, during the formation of benign and malignant tumours, the increase in the total proteasome pool occurs due to multiple immunoproteasome forms expressed at different ratios. These proteasomes include those containing all three immune subunits LMP7(β5i), LMP2(β1i), and LMP10(β2i); proteasomes containing the LMP7(β5i) immune subunit and the Y(β1) and Z(β2) constitutive subunits; and proteasomes containing the X(β5) constitutive subunit and the LMP2(β1i) and LMP10(β2i), immune subunits.

The decrease in chymotrypsin–like activity during tumour formation cannot be explained only by the change in the ratio of the X(β5) and LMP7(β5i) subunits responsible for that type of activity, since there is no correlation between those values (Table 1). It is likely that incorporation of the LMP2(β1i) subunit into proteasomes and/or intracellular regulation have more effect on the chymotrypsin–like activity.

In this study, we uncovered fundamental differences between proteasome pools in malignant and benign tumours. Of all the tumours studied, only hepatocellular carcinoma contained an increased amount of the 19S activator, which is present in the 26S proteasomes and controls their level (Fig. 2, Table 1). The increased level of the 26S proteasomes in hepatocellular carcinoma is easy to understand. High protein metabolism is typical for malignant tumours, including liver cancer [24, 25], which, in turn, requires more proteolytic enzymes, such as the 26S proteasomes.

The reason for the increased content of immunoproteasomes in hepatocellular carcinoma is not so clear, however. One can speculate that immunoproteasomes are expressed in the transforming cells so that the immune system can recognise and destroy those cells. It is possible that in our model some other links necessary for the immune reaction fail to function, and, regardless of the amount of immunoproteasomes generated in tumour cells, the immune system could destroy the cells. This is one issue we will study further. On the other hand, tumour cells may generate immunoproteasomes, which are known to possess an antioxidant function, in order to protect themselves from metabolites and other factors that would cause oxidative stress and apoptosis.

**Possible mechanism of immunoproteasome regulation in tumours**. The NO–dependent signalling pathway intended for quenching the oxidative stress in endotheliocytes causes additional expression of the LMP2(β1i) immune subunit to a larger extent than that of the LMP7(β5i) immune subunit [7], which coincides with our results on the expression dynamics of immune subunits during hepatic tumourogenesis (Table 1). The antioxidative function of immunoproteasomes in endotheliocytes is to eliminate the transferrin receptor and block free radical oxidation chain reactions involving Fe(II) [7]. It has been proven that NO acts as an antioxidant in malignant cells, too [26–28]. It is possible that immunoproteasomes in hepatocellular carcinoma participate in the NO–dependent signalling pathway that protects the tumour from oxidative stress. This hypothesis is confirmed by our data on the increased expression of nNOS in hepatocellular carcinoma (Fig. 2, Table 1), while there was little nNOS found in the control mouse liver. This result is in accord with data in the literature pointing to the fact that the amount of nNOS in adult mouse liver drops dramatically compared to that in foetal liver, where this enzyme regulates haematopoiesis [29].

**Fig. 3:**
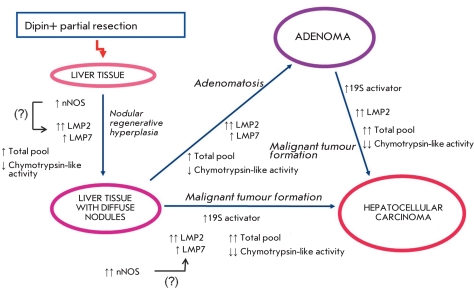
Schematic representation of liver cell malignant transformation based on the changes in the proteasome pool

In this study, we have shown that the formation of tumours in mouse liver is accompanied by significant changes in the proteasome pool. These changes are less pronounced in nodular hepatic hyperplasia and adenomatosis than in hepatocellular carcinoma. This suggests that nodular hepatic hyperplasia and adenomatosis may be stages preceding carcinogenesis. A schematic representation of a liver cell malignant transformation based on our results is given in Fig. 3.

Our results indicate that there is a need to identify the signalling pathways that change the expression of various proteasome subunits during tumourogenesis. In addition, we can conclude that the 19S proteasome activator overexpressed in malignant tumours can be a potential target for the development of new anticancer drugs. At the moment, the first proteasome inhibitor anticancer drug, Bortezomib (Velcade), is being used clinically [30]. Bortezomib is injected into a patient’s bloodstream, and it is administered along with other anticancer medication. Bortezomib, a boronic acid derivative, selectively inhibits the chymotrypsin–like activity of all proteasome forms and temporarily induces apoptosis, primarily of neoplastic cells. The prolonged inhibition of proteasome activity, however, induces feedback mechanisms and the generation of new proteasomes [31]; hence the drug’s temporary therapeutic effect. At the same time, Bortezomib affects the total proteasome pool in all organs, thus causing side effects such as fatigue, atony, gastrointestinal disorders, peripheral neuropathy, and significant deterioration of the general wellbeing of patients [30]. In this regard, suppressing the functions of the 19S activator while maintaining the proteasome’s proteolythic activity appears to be a more efficient and safer approach to anticancer therapy.

## CONCLUSIONS

The formation of hepatic nodular regenerative hyperplasia, adenomatosis, and carcinoma is accompanied by changes in the proteasome pool, the changes having similarities, as well as differences. The similarities are the increase in the content of immunoproteasomes and in the total proteasome pool, and the decrease in the proteasome chymotrypsin–like activity in all tumour types compared to the control samples. The difference is in the behavior of the 19S proteasome activator content, which is increased only in hepatocellular carcimona.

The dynamics of changes in the proteasome pools in liver with diffuse nodules, adenoma, and carcinoma indicates that nodular regenerative hyperplasia and adenomatosis may be stages preceding carcinogenesis.

The 19S proteasome activator, which is overexpressed only in malignant tumours, can be a promising target for the development of new anticancer drugs.

## ABBREVIATIONS

nNOS: neuronal nitric oxide synthase
dipin: 1,4–Bis–[N,N’–di(Ethylene)–phosphamide]piperazine
Suc–LLVY–AMC: N–succinyl–leu–leu–val–tyr7–amido–4–methyl coumarin
MG132: Z–leucyl–leucyl–leucinal, mAb – monoclonal antibody
pAb: polyclonal antibody
